# Draft Genome Sequence of a Clinical Isolate, *Aeromonas hydrophila* SNUFPC-A8, from a Moribund Cherry Salmon (*Oncorhynchus masou masou*)

**DOI:** 10.1128/genomeA.00133-12

**Published:** 2013-02-07

**Authors:** Jee Eun Han, Ji Hyung Kim, Casiano Choresca, Sang Phil Shin, Jin Woo Jun, Se Chang Park

**Affiliations:** a Laboratory of Aquatic Biomedicine, College of Veterinary Medicine and Research, Institute for Veterinary Science, Seoul National University, Seoul, Republic of Korea; b Korea Institute of Ocean Science & Technology, Ansan, Republic of Korea

## Abstract

We present the genome of a clinical isolate, *Aeromonas hydrophila* SNUFPC-A8, from a moribund cherry salmon. The completed draft genome of this strain shows high sequence homology to the reference strain *A. hydrophila* ATCC 7966 (NC008570.1) and known plasmids pAsa2 and pAAk1 from other *Aeromonas* species (NC004925.1 and NC019014.1).

## GENOME ANNOUNCEMENT

In aquatic environments, fish are predisposed to *Aeromonas* infections because of the widespread distribution of these pathogens and the stress induced by intensive culture (1). In humans, motile *Aeromonas* species have received increasing attention as emergent agents of food-borne gastrointestinal disease. Recently, *Aeromonas* was reported as the cause of necrotizing fasciitis, better known as flesh-eating bacteria, especially in patients with immunosuppression who underwent an aquatic wetting (2).

The complete genome of *A. hydrophila* ATCC 7966 (NC008570.1), a well-characterized type strain isolated from a tin of milk, was sequenced previously (3). However, there are no genome sequences available for *A. hydrophila* strains isolated from clinical samples.

The *A. hydrophila* strain SNUFPC-A8 isolated from a kidney of a moribund cherry salmon (*Oncorhynchus masou masou*) (4) was used for draft-genome sequencing. Genomic DNA was extracted (5) and sequenced using the Roche/454 GS FLX titanium pyrosequencing method with 37.5× coverage (Macrogen, South Korea). Putative open reading frames (ORFs) were predicted by using Glimmer 3.0 software (6), and the putative functions of the ORFs were determined using the BLAST program in GenBank (7).

The sequence data consisted of a total of 185,951,609 bp and 303,268 reads with an average read length of 613.16 bp. Furthermore, the data include 295,118 assembled reads and 3,418 partially assembled reads. Using *de novo* software (v. 2.6), the reads were assembled into 59 contigs, which included 41 contigs that were longer than 500 bp. The average contig size was 13,019 bp, and the data include 300 singletons. The draft genome of *A. hydrophila* SNUFPC-A8 was 4,969,090 bp in length, and a total of 4,779 ORFs were discovered.

Gene ontology (GO) searches were performed using all predicted ORFs, and the results revealed that 35%, 33%, and 11% of the sequences included genes related to biological processes, molecular functions, and cellular components, respectively. Of the GO categories related to biological processes, metabolic processes represented the most dominant category, which contained 32% of genes. In the cellular component category, 45% of the genes were unclassified. Based on molecular functions characterized by GO terms, 40% of the genes were associated with catalytic activities.

The strain SNUFPC-A8 shows high nucleotide sequence similarity to the reference strain *A. hydrophila* ATCC 7966 (NC008570.1), and 217,553 reads of SNUFPC-A8 were fully aligned. Additionally, SNUFPC-A8 shows sequence homology with the known plasmids *A. salmonicida* A449 and *A. aquariorum* AAK1 (NC004925.1 and NC019014.1) The sequence data generated in this study will contribute to the understanding of genome diversity of *A. hydrophila* and other *Aeromonas* species.

### Nucleotide sequence accession number.

The nucleotide sequence for the draft genome was deposited in GenBank under accession number AMQA00000000.
